# Harnessing eco-friendly synthesis: the in vitro bioactivity and biocompatibility of ceramic spinels

**DOI:** 10.1038/s41598-026-50766-x

**Published:** 2026-05-11

**Authors:** Sayed H. Kenawy, Gehan T. El-Bassyouni, Esmat M.A. Hamzawy, Mahmoud T. Abo-Elfadl, H. K. Abd El-Hamid

**Affiliations:** 1 Refractories, Ceramics and Building Materials Department, National Research Centre (NRC), 33 El-Buhouth St., Dokki, Cairo, 12622 Egypt; 2 Glass Research Department, National Research Centre (NRC), 33 El- Buhouth St., Dokki, Cairo, 12622 Egypt; 3 Cancer Biology and Genetics Laboratory Centre of Excellence for Advanced Sciences, National Research Centre (NRC), 33 El-Buhouth St., Dokki, Cairo, 12622 Egypt

**Keywords:** MnAl_2_O_4_ spinel, ZnAl_2_O_4_ spinel, Microstructure, Bioactivity, Cytotoxicity, Biotechnology, Materials science

## Abstract

Harnessing a sustainable, starch-assisted gel synthesis, researchers have engineered MnAl₂O₄ and ZnAl₂O₄ spinel ceramics, calcined at 1000 °C, as next-generation bioactive materials. Comprehensive characterization via XRD and FTIR confirmed phase-pure crystallinity, while advanced FE-SEM/EDX and DLS analyses unveiled a pivotal divergence in their physical landscapes: MnAl₂O₄ emerged with a distinctive nanoscale architecture and a richer surface hydroxyl population compared to its ZnAl₂O₄ counterpart. This fundamental difference in surface chemistry and morphology directly translated to superior performance in simulated physiological environments. During in vitro bioactivity tests in simulated body fluid, both ceramics fostered essential calcium-phosphate layers, yet MnAl₂O₄ demonstrated markedly accelerated apatite nucleation, achieving a Ca/P ratio nearing that of ideal bone mineral (stoichiometric hydroxyapatite), alongside enhanced microstructural densification. Mechanically, it exhibited a robust, time-dependent increase in compressive strength, outperforming ZnAl₂O₄. Critically, biocompatibility assessment on human dermal fibroblasts revealed that MnAl₂O₄ maintained excellent cytocompatibility across all concentrations and exposure periods. In contrast, ZnAl₂O₄ induced mild, time-dependent cytotoxic effects. Collectively, these findings position MnAl₂O₄ spinel not merely as a compatible material, but as a highly promising and bioactive ceramic candidate poised to advance the field of bone graft substitutes and orthopedic implants.

## Introduction

In recent years, mixed ceramic oxides have emerged as a critically important class of materials, garnering significant attention across the scientific community. This interest stems from their exceptional and versatile suite of properties, including remarkable optical, catalytic, electronic, and mechanical characteristics, which make them suitable for a vast array of advanced applications. Consequently, research into spinels spans diverse disciplines, from fundamental materials physics and electronics to applied optics, structural mechanics, and increasingly, the field of biomedicine^1,2^.

Within this broad family, spinel-structured aluminates, with the general formula MAl₂O₄ (where M represents a divalent cation such as Mg²⁺, Zn²⁺, or Mn²⁺), have become a subject of focused investigation. These compounds are particularly prized for their outstanding chemical stability, high mechanical robustness, and highly tunable physicochemical properties, which can be tailored through cation selection and synthesis methods. A prominent member of this group is manganese aluminate (MnAl₂O₄). It crystallizes in the normal spinel structure (space group *Fd–3 m*), wherein Mn²⁺ cations occupy the tetrahedral sites and Al³⁺ cations reside in the octahedral coordination spheres within the cubic close-packed oxygen lattice^3^. This specific atomic arrangement is fundamental in dictating its resultant optical, magnetic, and surface properties, making it a compelling candidate for specialized applications where its unique ionic configuration can be exploited.

While manganese has been established as a beneficial element in biomaterials, the scientific focus has predominantly centered on manganese-doped composite systems, such as bioactive glasses and ceramics, rather than exploring pure manganese aluminate spinels as standalone biomaterials. For instance, research has demonstrated that Mn-doped bioactive glass nanoparticles not only facilitate the formation of apatite-like crystals in simulated body fluid (SBF) but also provide sustained manganese release coupled with antimicrobial properties^4^. Beyond bioactivity, incorporating manganese ions into silicate-based bioactive glasses has shown promising biocompatibility and, significantly, the potential to upregulate osteoblastic gene expression, including key markers like alkaline phosphatase (ALP) and bone morphogenetic proteins (BMPs), thereby actively promoting bone regeneration^5^.

The utility of manganese extends into the broader domain of nanotechnology. Manganese-based nanomaterials are widely regarded as environmentally benign, cost-effective, and biocompatible agents, often celebrated for their excellent adsorption capabilities. This unique combination of properties underpins their suitability for a diverse spectrum of advanced biomedical applications. These range from foundational roles in targeted drug delivery and biosensing to active therapeutic functions in anticancer and antimicrobial therapies, antioxidant activity, nanozyme catalysis, and photothermal treatment^6–9^.

In contrast to the limited exploration of pure manganese spinels, zinc aluminate (ZnAl₂O₄) has garnered notable interest within a specific biomedical niche: surface coatings for metallic implants, particularly on titanium. Research has demonstrated that coatings incorporating ZnAl₂O₄, often applied via techniques like micro-arc oxidation (either as a pure phase or in composite with ZnO), can induce the formation of bone-like mineral clusters when immersed in simulated body fluid (SBF), signaling promising bio-mineralization potential^10^. This property aligns with the critical biological role of zinc as an essential trace element, vital for bone formation and metabolism. Zinc is a known cofactor for the enzyme alkaline phosphatase (ALP), a key player in bone matrix mineralization, and contributes to various other metabolic processes essential for tissue health^11–13^. However, a crucial and well-documented challenge emerges: the bioactivity and biocompatibility of these systems are highly dependent on the precise concentration and chemical state of zinc. Studies reveal a delicate balance; while a certain zinc loading promotes mineralization, excessive ZnO content in coatings can paradoxically inhibit apatite nucleation. More critically, cell viability assays using osteoblast-like cells have shown that high zinc concentrations can lead to cytotoxicity over time, underscoring the imperative to optimize zinc release profiles for safe and effective application^10^.

This dichotomy positions zinc as a “double-edged sword” biologically essential yet potentially toxic, highlighting the need for material designs that ensure its controlled and sustained delivery. Interestingly, the fundamental spinel crystal structure of ZnAl₂O₄ itself may offer a sophisticated solution to this release-control problem. Research in environmental science has shown that zinc ions can be effectively immobilized within the stable ZnAl₂O₄ lattice during high-temperature synthesis, drastically reducing their leachability compared to more soluble zinc compounds. This inherent property suggests that ZnAl₂O₄ could act not merely as a passive coating component but as an intelligent, controlled-release matrix for Zn²⁺ ions in physiological environments. By stabilizing zinc within its crystalline framework, the spinel could theoretically modulate ion release to remain within the therapeutic window sufficient to elicit beneficial osteogenic and antibacterial effects^14^, while avoiding cytotoxic thresholds. Yet, this hypothesized behavior remains largely unexplored in biologically relevant models, representing a significant gap between materials science and practical biomedicine^15,16^.

This study explores the largely unexamined bioactive potential of stable mixed-oxide ceramics composed of biologically relevant elements. The true novelty of this work resides in its deliberate and controlled integration within a single, unified comparative framework. By developing an eco-friendly hydrolyzed gel route that yields phase-pure MnAl₂O₄ and ZnAl₂O₄ spinels at remarkably low processing temperatures, we not only advance energy-efficient and scalable ceramic synthesis, but more importantly, we create a platform for direct, side-by-side evaluation under identical experimental conditions. This methodological rigor allows us to isolate the intrinsic influence of cation chemistry (Mn²⁺ versus Zn²⁺) from extraneous processing variables for the first time. Both spinels were synthesized under identical conditions and comprehensively characterized in terms of their structural and morphological features using XRD, FTIR, and FE-SEM/EDX. Their basic biocompatibility was evaluated through cytotoxicity testing in accordance with ISO 10993-5, while their bone-bonding ability was investigated by monitoring hydroxycarbonate apatite (HCA) formation in simulated body fluid. Overall, this work provides a critical assessment of these spinel ceramics, effectively linking their sustainable synthesis to standardized biomaterial evaluation protocols.

## Materials and methods

### Raw materials

The raw chemical precursors for this study were Zinc Acetate Hexahydrate (Sigma-Aldrich), Manganese Acetate Tetrahydrate (Merck), and Aluminum Chloride Hexahydrate (Merck). To facilitate the synthesis, commercial corn starch was employed as a biogenic gelling agent. Corn starch is an abundant, renewable, and low-cost polysaccharide characterized by a high density of hydroxyl (–OH) functional groups. When heated in an aqueous medium, its long-chain amylose and amylopectin molecules undergo gelatinization, a process of water absorption, swelling, and thickening that results in a viscous gel matrix. This unique behavior, combined with its intrinsic biocompatibility, solubility, and high recrystallization stability, makes it an excellent, environmentally benign candidate for producing nanoscale ceramics. The gel network effectively homogeneously disperses the metal cations, inhibits premature particle agglomeration, and can be cleanly removed during subsequent calcination, aiding in the formation of phase-pure zinc aluminate (ZnAl₂O₄) and manganese aluminate (MnAl₂O₄) spinels with controlled morphology.

### Synthesis of zinc aluminate spinel

Zinc aluminate spinel (ZnAl₂O₄) was synthesized via a starch-assisted gel method. First, a homogeneous starch gel was prepared by dispersing 20 g of commercial corn starch in 100 mL of hot distilled water under vigorous stirring until a clear, viscous gel formed^17^. Separately, precursor solutions were prepared. A zinc acetate solution was made by dissolving 0.01715 mol of Zinc Acetate Hexahydrate in 50 mL of distilled water with 30 min of stirring. An aluminum chloride solution was prepared similarly by dissolving 0.0207 mol of Aluminum Chloride Hexahydrate in 50 mL of distilled water.

These precursor solutions were then combined: the zinc acetate solution was added to the aluminum chloride solution in a 1:1 molar ratio with continuous stirring to ensure a homogeneous mixture. This combined metal solution was subsequently poured into the prepared starch gel and stirred thoroughly until gelation occurred. The final gel was aged by refrigeration at 4 °C for 24 h to complete the gelling process. The aged gel was then transferred to an electric furnace for calcination. It was heated to 1000 °C at a controlled ramp rate of 3 °C/min and held at this temperature for 1 h to facilitate solid-state reaction and crystallize the final ZnAl₂O₄ spinel powder.

### Synthesis of manganese aluminate spinel

To unlock the bioactive potential of manganese, we synthesized the manganese aluminate spinel (MnAl₂O₄) using our established, eco-friendly starch-gel protocol. This method ensured direct comparability with its zinc counterpart. A precise stoichiometric blend was achieved by combining 0.0204 mol of Manganese Acetate Tetrahydrate with 0.0207 mol of Aluminum Chloride Hexahydrate in aqueous solution. This precursor mixture was then incorporated into a renewable corn starch gel matrix, which acts as a nano-template to control morphology and prevent agglomeration. The gelled composite was aged, then subjected to a controlled thermal transformation. Through calcination at 1000 °C/1 h (using a consistent ramp rate of 3 °C/min**)**, the organic template was cleanly removed, triggering solid-state diffusion and crystallizing the final, phase-pure MnAl₂O₄ powder. This synthesis pathway highlights a green, reproducible route to obtain this underexplored ceramic for advanced biomedical evaluation.

### Material characterizations

The structural and chemical identity of the synthesized spinels was rigorously elucidated through a comprehensive suite of advanced characterization techniques. Phase purity and crystal structure were confirmed via X-ray diffraction (XRD) using a Bruker D8 ADVANCE diffractometer with Cu-Kα radiation (λ = 0.15418 nm, 45 kV, 40 mA). High-resolution patterns were acquired over a 2θ range of 5°–70° with a precise step size of 0.02° and a counting time of 2 s per step under ambient conditions. Molecular bonding and functional groups were probed by Fourier-transform infrared spectroscopy (FTIR) in reflectance mode on a JASCO FT/IR-4600 spectrometer (Asia Portal), collecting detailed spectra from 400 to 4000 cm⁻¹ with a resolution of 2 cm⁻¹ at room temperature (20 °C). Particle size distribution in suspension was determined by dynamic light scattering (DLS) using a Malvern Zeta-sizer Nano ZS system with a 633 nm He/Ne laser at a 173° backscattering angle. Finally, ultra-high-resolution morphology and elemental composition were visualized and verified using field-emission scanning electron microscopy coupled with energy-dispersive spectroscopy (FE-SEM/EDS) on an Inspect S50 instrument (FEI Co., Japan; Model T810, Serial D8571), enabling nanoscale investigation at magnifications up to 300,000×.

### In vitro biological examination

The bioactivity of the synthesized ceramics was first assessed using the well-established SBF immersion test, a widely recognized preliminary indicator of bone-bonding potential. While we acknowledge that SBF alone cannot confirm true in vivo osseointegration or clinical readiness, it served here as an essential screening tool following *Kokubo’s* protocol to evaluate early surface reactivity and apatite formation^18^. To ensure a robust and multidimensional evaluation, we complemented SBF analysis with comprehensive assessments of bulk density, porosity, compressive strength over time (1–28 days), post-immersion surface chemistry (FTIR, FE-SEM/EDX), and cytocompatibility. Together, these integrated approaches provide a holistic view of the materials’ structural integrity, biological safety, and bioactive behavior.

According to ISO/FDIS guidelines, disc-shaped samples from each of the three batches were carefully positioned in sterile, non-reactive polypropylene containers. The experiment was conducted in Simulated Body Fluid (SBF), a solution ionically analogous to human blood plasma under controlled physiological conditions at 37 ± 0.5 °C^19^. To ensure consistency, the volume of SFB in each container was precisely calculated using a standard volume-to-surface-area ratio of 10 mL per cm², based on the measured sample surface area (Sa) as defined by Eq. (1):

1 $${\rm V_{SBF} = Sa/10 [mL]}$$

Sealed containers were incubated for 1, 3, 7, 14, 21, or 28 days. After immersion, samples were removed, rinsed with deionized water to halt reactions and remove residual SBF, air-dried at room temperature, and analyzed via XRD, FTIR, and FE-SEM/EDX. These analyses evaluated the formation and distribution of surface calcium phosphate phases (characteristic of hydroxyapatite), which are indicative of bioactivity^20^.

To comprehensively evaluate the materials’ performance, a multifaceted characterization protocol was implemented. Chemical bioactivity indicators were tracked by monitoring the evolution of the simulated body fluid (SBF) environment. Changes in pH were recorded with a precision electrolyte-type pH meter, while the concentrations of critical calcium (Ca), phosphorus (P), manganese (Mn), and zinc (Zn^2+^) ions released into solution were quantified using high-sensitivity inductively coupled plasma optical emission spectroscopy (ICP-OES; Agilent 5100VDV)^21^.

Concurrently, the key mechanical and physical properties essential for a structural biomaterial were assessed. Compressive strength was determined following the ASTM C773-88(2020) standard, using a Lloyd Instrument (Model LR 10 K) to test cylindrical specimens (Ø12 mm × 19 mm height) fabricated under a uniform pressure of 25 kN/mm²^22^. The testing conditions were as follows: a loading rate of 0.5 mm/min, three replicates per sample, and a brittle fracture failure mode, which is typical of porous ceramic. Furthermore, the bulk density and apparent porosity of the sintered ceramics were precisely measured at ambient temperature using the classic Archimedes’ principle with water as the immersion medium^23^. This integrated approach provides a complete picture of the materials’ interaction with a physiological environment and their structural integrity.

### Cytotoxicity test

#### Cell culture

Using normal human dermal fibroblasts (HDFs) instead of osteogenic models for evaluating bone-targeted materials is not a compromise, it is a deliberate. Fibroblasts are exquisitely sensitive, non-mineralizing sentinels; their robust viability on MnAl₂O₄ delivers clean, interpretable proof of non-toxicity before any investment in costly, variable osteoblast or in vivo studies. In this study, primary neonatal HDFs (ATCC, USA) were employed specifically as an initial cytotoxicity screening tool to verify the absence of leachable toxic species. As per ISO 10,993 guidelines, the MTT assay on normal fibroblasts is a well-established, widely accepted method for preliminary biocompatibility assessment^24^. Importantly, fibroblasts are among the first cells to encounter an implanted biomaterial, making them a sensitive and clinically relevant indicator of cytocompatibility and ensuring a favorable response is essential to confirm safety for surrounding healthy tissues, a critical prerequisite for any bone regeneration application. Cells were cultured in Basal Fibroblast Medium (10% FBS, 2 mM L-glutamine, 1% antibiotic-antimycotic; Biowest, France) under standard conditions (37 °C, 80% humidity, 5% CO₂). Sub-culturing involved triple washing with DPBS (Biowest, France), trypsinization with 0.25% trypsin-EDTA (Serana Europe, Germany), followed by full medium dispersion, centrifugation, and counting^25^. The authors do not claim osteoinduction, only bioactivity and biocompatibility, and for these foundational endpoints, HDFs are not merely appropriate; they are the optimal first-line model.

To ensure full transparency and compliance with ISO 10993-5 and ISO 10993-12, we explicitly confirm that the indirect (extract) method was employed a deliberate choice that isolates leachable toxicity from confounding surface topography or mechanical interference. Briefly, sterilized MnAl₂O₄ and ZnAl₂O₄ ceramics were extracted in serum-free Basal Fibroblast Medium (6 cm²/mL, 37 °C, 24 h, 60 rpm), followed by filtration (0.22 μm) to remove particulates. Normal human neonatal dermal fibroblasts (ATCC, USA) were seeded at 1 × 10⁴ cells/well, exposed to material extracts for 24, 48, and 72 h, and assessed via MTT assay, with fresh medium as a negative control and 0.1% SDS as a positive control. The indirect method was selected for two strategic reasons: (i) it enables precise, quantitative detection of leachable toxic species without physical disruption of the cell monolayer, and (ii) it represents the ISO-preferred first-line screen for novel ceramics, where particle shedding or surface roughness could otherwise produce artefactual direct-contact outcomes. All experiments were performed in triplicate with three independent replicates.

#### Cell proliferation activity

Cell proliferation of the tested samples was measured relative to HDFn cells after 1, 3, and 5 consecutive days using the MTT Cell Viability Assay.

Briefly, Cells (1 × 10^4^ cells/ well) were plated in a flat-bottom 96-well microplate and treated with 20 µl of the samples to obtain final concentrations 100, 50, 25, 12.5, 6.25, and 3.125 µg/mL of the tested samples for 1, 3, and 5 days at 37º C, in a humidified 5% CO_2_ atmosphere. After each incubation, the medium was removed, and 40 µL MTT solution was added / well and incubated for an additional 4 hours. MTT crystals were solubilized by adding 180 µL of acidified isopropanol / well, and the plates were shaken at room temperature, followed by photometric determination of the absorbance at 570 nm using a microplate ELISA reader. Three repeats were performed for each concentration, and the average was calculated. Data were expressed as the percentage of relative viability compared with untreated cells^26,27^.

#### Alkaline phosphatase activity

The ALP activity assay was performed using the Alkaline Phosphatase Assay Kit (Fluorometric) (ab83371) (Abcam, Waltham, MA, USA) according to the manufacturer’s protocol on the supernatant of the HDFn cells after treatment with 50 µg/mL for 3 and 5 days in comparison to the respective untreated control cells. The fluorescence signal was measured at Ex/Em = 360 nm/440 nm using a microplate reader, FLUOstar OPTIMA (BMG LABTECH GmbH, Ortenberg, Germany)^28^.

#### Microscopic cell morphology changes

To evaluate cellular response, morphological changes were assessed using complementary microscopy techniques. Initial gross morphological analysis was performed using an inverted light microscope (Olympus CKX41 equipped with an SC180 camera). For detailed visualization of apoptotic events, cells were stained with acridine orange/ethidium bromide (AO/EtBr) and examined under a fluorescent microscope (Zeiss AxioImager Z2 with an AxioCam MRc3 camera) to trace specific alterations in cell membranes and nuclei^29^.

### Statistical analysis

The results were derived from independent experiments and are expressed as mean ± standard deviation (SD) with a sample size of *n* = 3. Statistical analysis was carried out using two-way ANOVA followed by Tukey’s post-hoc test, with *p* < 0.05 considered statistically significant. The SD values are included in each table, and the error bars in the figures correspond to these standard deviations.

## Results and discussion

### Materials characterization

**Fig. 1:**
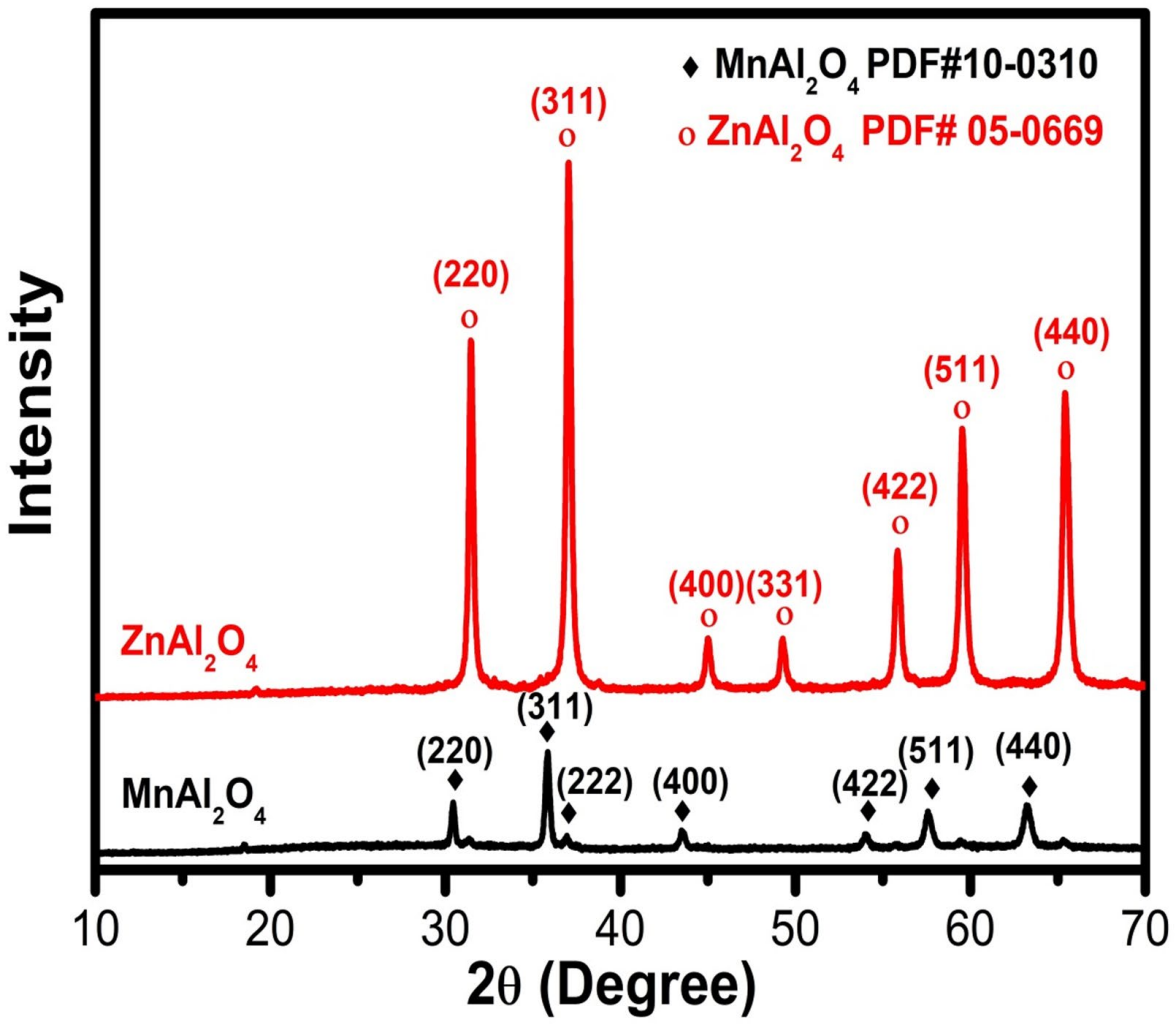
XRD pattern of MnAl_2_O_4_ and ZnAl_2_O_4_ spinels sintered up to 1000℃.

Figure 1 presents the X-ray diffraction (XRD) patterns for the synthesized MnAl₂O₄ and ZnAl₂O₄ powders, which unequivocally confirm the successful formation of highly crystalline, phase-pure spinel structures. The sharp, well-defined diffraction peaks for both materials indicate excellent crystallinity. For MnAl₂O₄, all observed peaks correspond exclusively to the cubic spinel structure of galaxite, showing perfect alignment with the reference pattern PDF# 10–0310^30^. Similarly, the XRD profile for ZnAl₂O₄ is indexed entirely to the cubic spinel phase of gahnite, matching the standard reference PDF# 05-0669^31^ without any detectable secondary or impurity phases. These results validate the efficacy of the starch-assisted gel synthesis and subsequent calcination protocol in producing single-phase MnAl₂O₄ and ZnAl₂O₄ ceramics with the intended crystal structures.

**Fig. 2:**
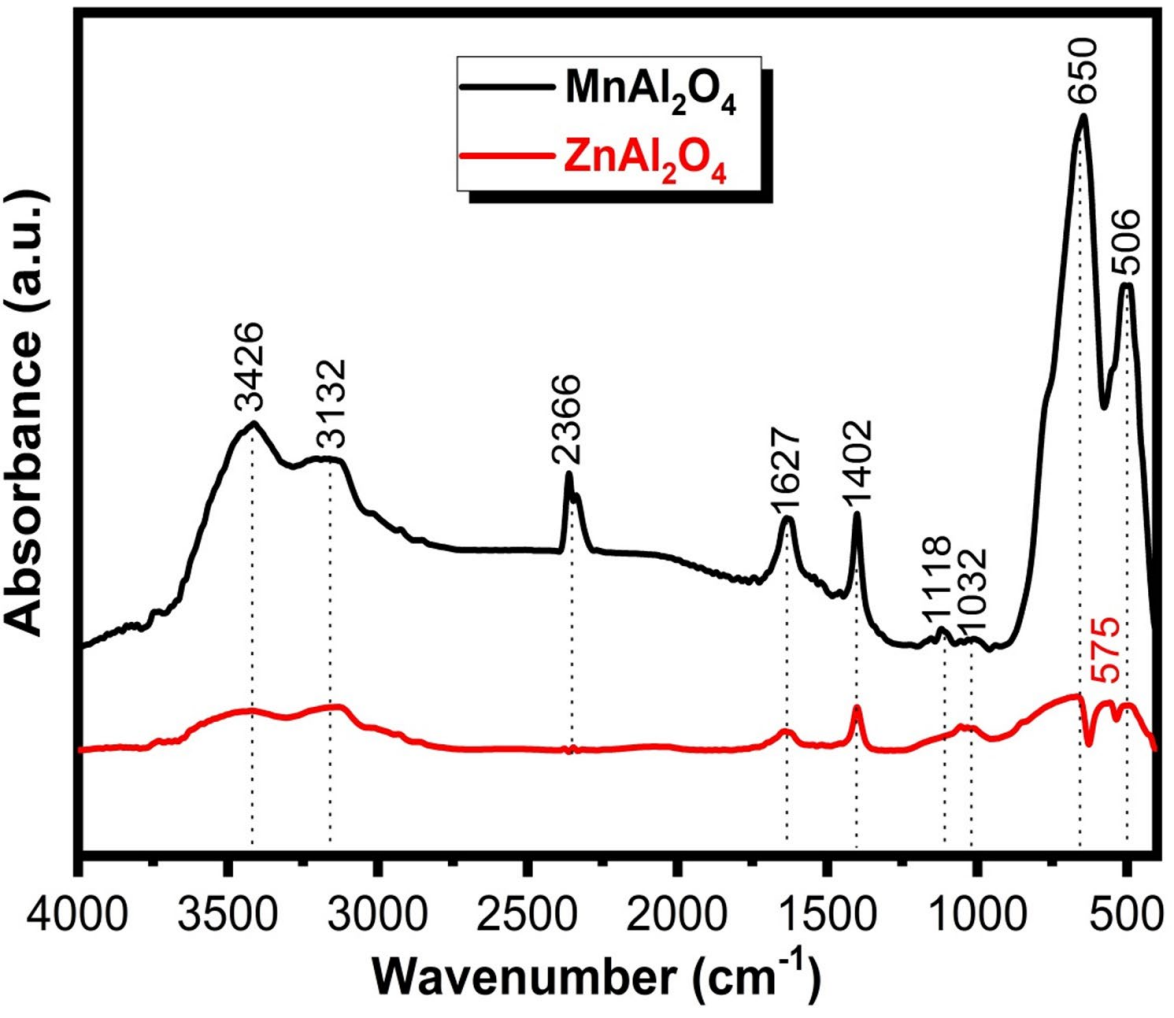
FTIR spectra of MnAl_2_O_4_ and ZnAl_2_O_4_ spinels sintered up to 1000 °C.

The FTIR spectra in Fig. 2 provide compelling molecular-level evidence for the successful formation of both spinel structures while revealing a striking contrast in their surface chemistry. The fingerprint region unequivocally confirms the characteristic metal-oxygen (M–O) framework, with distinct bands at 506, 575, 650, 1032, and 1118 cm⁻¹ corresponding to stretching and bending vibrations within the tetrahedral and octahedral sites of the spinel lattice^32,33^. Beyond the lattice vibrations, the spectra unveil a critical difference in surface properties. MnAl₂O₄ displays pronounced broad bands at 3426 and 3132 cm⁻¹, accompanied by a bending mode at 1627 cm⁻¹, which collectively indicate a significantly higher concentration of surface-adsorbed water and hydroxyl groups compared to ZnAl₂O₄. The zinc aluminate spinel shows only weak features in this region, pointing to a comparatively more hydrophobic surface^34^. Minor bands at 1402 cm⁻¹ and 2366 cm⁻¹ (the latter more prominent in MnAl₂O₄) are assigned to trace carbonate species or adsorbed atmospheric CO₂^35^.

Overall, the analysis confirms both materials possess the targeted, well-defined spinel architectures, while simultaneously highlighting that MnAl₂O₄ presents a more hydrophilic and hydrated surface prior to biological testing, a fundamental characteristic that may profoundly influence its subsequent bio-interactivity.

**Fig. 3:**
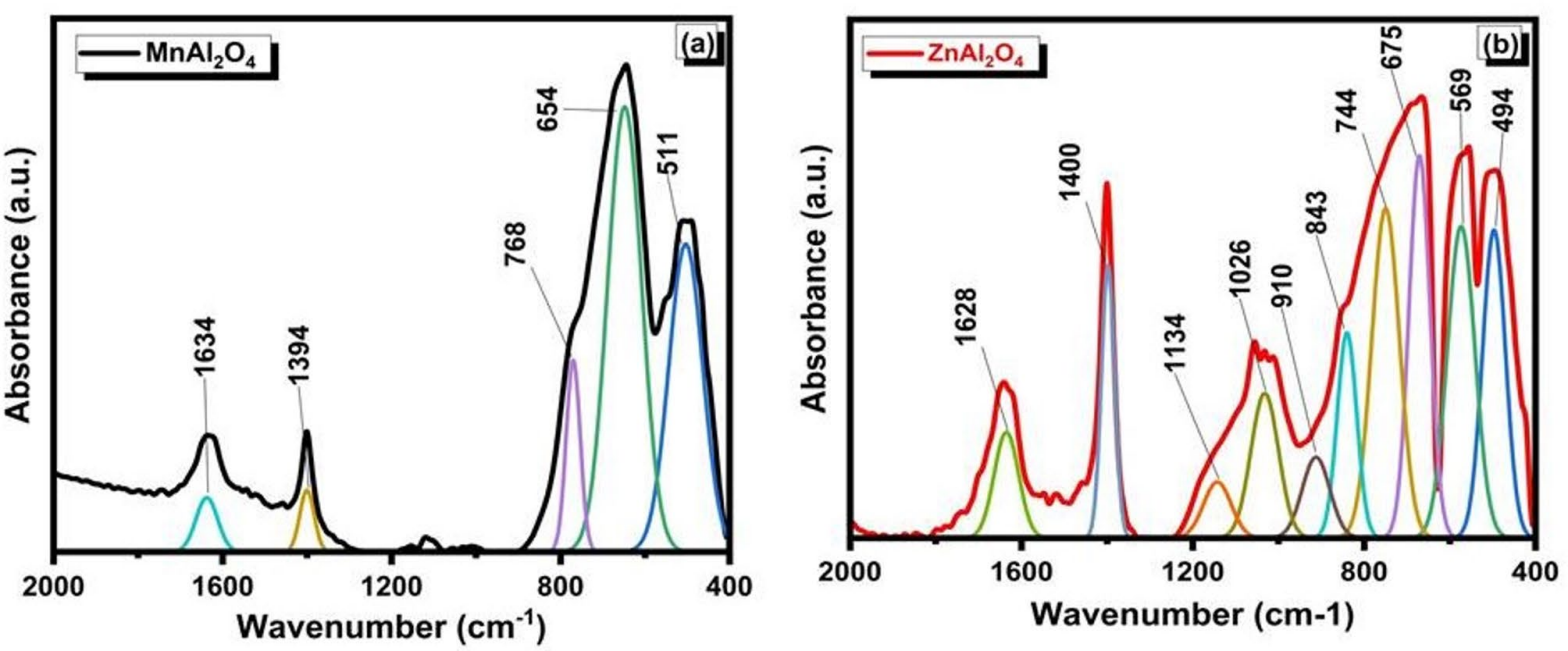
Deconvoluted FTIR spectra of (**a**) MnAl₂O₄ and (**b**) ZnAl₂O₄ spinels sintered up to 1000 °C.

Figure 3 presents the deconvoluted FTIR spectra of **(a)** MnAl₂O₄ and **(b)** ZnAl₂O₄ powders before immersion, highlighting the vibrational bands associated with their spinel structures. In the MnAl₂O₄ spectrum, the prominent bands observed in the low-wavenumber region at around ~ 768, ~654, and ~ 511 cm⁻¹ are characteristic of metal–oxygen (M–O) stretching vibrations within the spinel lattice. These bands are mainly attributed to Al–O vibrations in octahedral sites and Mn–O vibrations in tetrahedral sites, confirming the formation of a normal/inverse spinel structure. The weak bands appearing at higher wavenumbers (~ 1634 and ~ 1394 cm⁻¹) are assigned to adsorbed water bending vibrations and residual carbonate species from synthesis or atmospheric exposure. For ZnAl₂O₄, the deconvoluted spectrum shows multiple well-resolved bands, indicating a more complex vibrational environment. The strong absorption bands in the range of ~ 400–750 cm⁻¹ (notably at ~ 494, ~569, ~ 675, and ~ 744 cm⁻¹) correspond to Zn–O and Al–O stretching modes in tetrahedral and octahedral coordination, which are typical fingerprints of the ZnAl₂O₄ spinel phase. Additional bands at higher wavenumbers (~ 910–1134 cm⁻¹ and ~ 1400–1628 cm⁻¹) are related to Al–O vibrations with lattice distortions and to surface-adsorbed hydroxyl groups or carbonate residues^33–35^.

The deconvoluted FTIR analysis confirms the successful synthesis of both spinels and reveals critical differences in their structure and surface chemistry. The spectra provide distinct fingerprints for the Mn–O and Zn–O coordinations, with ZnAl₂O₄ exhibiting greater vibrational complexity, suggesting a more distorted lattice. Importantly, detected surface hydroxyls and carbonates define the initial interface of each material, a key factor that will govern their subsequent interaction and bioactive behavior in a physiological environment.

**Table 1:** Dynamic light scattering (DLS) of the prepared spinels samples.

Sample	Hydrodynamic diameter (H_D_, nm)	Polydispersity index (PDI)
MnAl_2_O_4_	83.80 *±* 16.35	0.462
ZnAl_2_O_4_	484.2 *±* 25.24	0.625

**Fig. 4:**
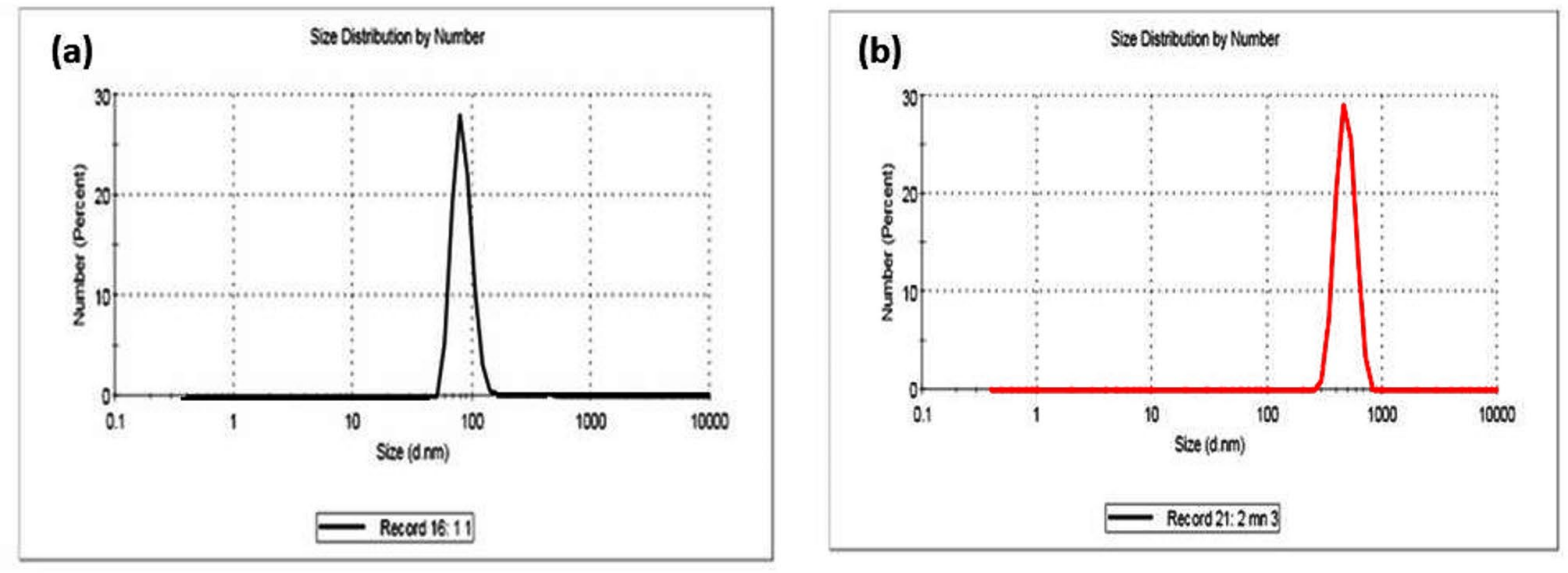
Dynamic light scattering (DLS) of the prepared (**a**) MnAl_2_O_4_ and (**b**) ZnAl_2_O_4_ spinel samples.

Dynamic light scattering (DLS) analysis revealed a striking contrast in the particle characteristics of the two synthesized spinels, with direct implications for their biological interaction. As summarized in Table 1 and visualized in Fig. 4a–b, the MnAl₂O₄ spinel formed notably nano-scale particles, exhibiting a hydrodynamic diameter of 83.80 ± 16.35 nm and a polydispersity index (PDI) of 0.462, which denotes a moderately uniform and well-dispersed population. In sharp contrast, the ZnAl₂O₄ particles were significantly larger, with an average hydrodynamic diameter of 484.2 ± 25.24 nm and a higher PDI of 0.625, indicating a broader, more heterogeneous size distribution. This pronounced divergence in particle size and dispersity underscores fundamental differences in the synthesis outcome, suggesting that MnAl₂O_4_ may offer a more favorable nano-morphology for biomedical applications where surface area and consistency are critical.

**Fig. 5:**
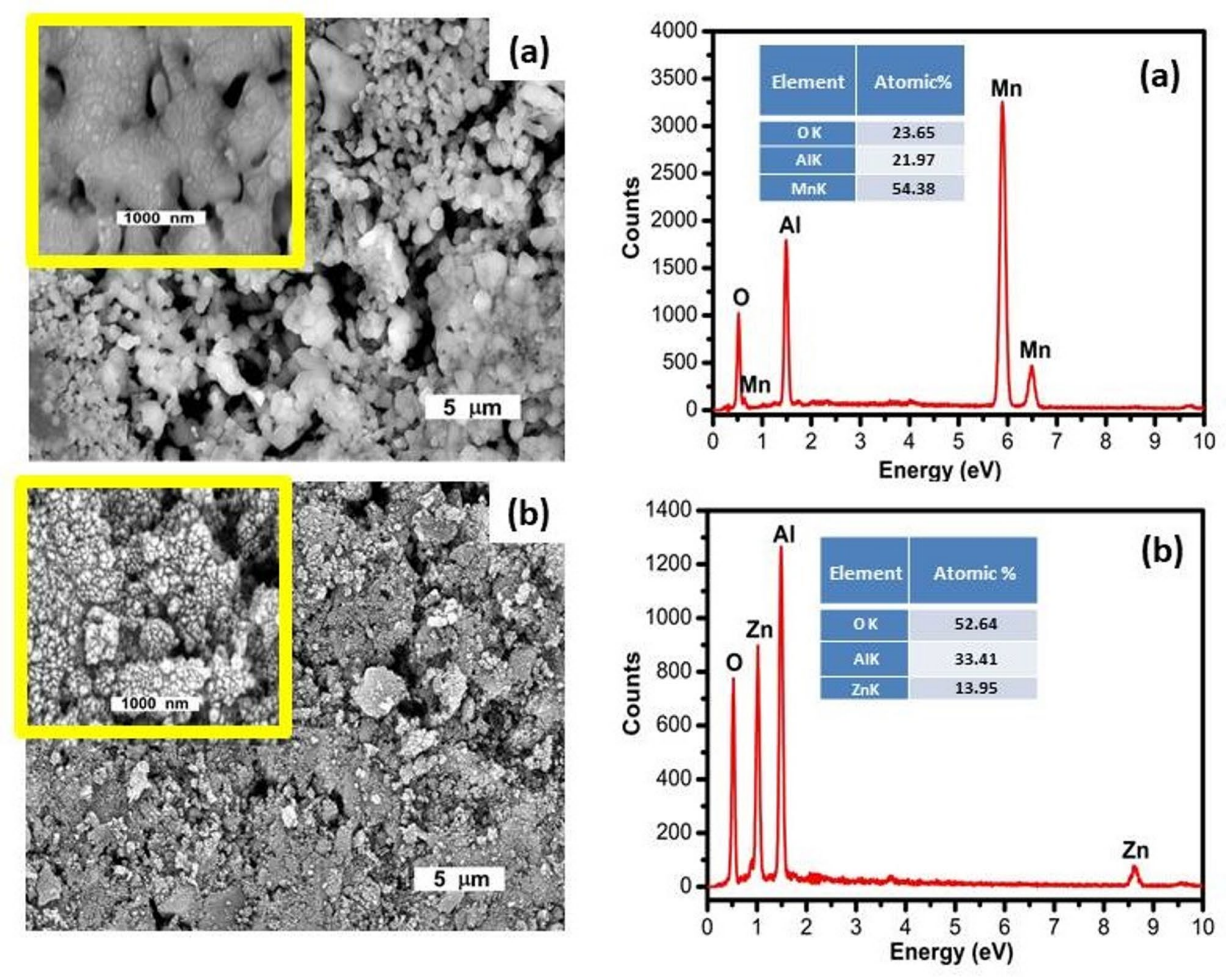
SEM micrographs and EDX of the synthesized (**a**) MnAl₂O₄ and (**b**) ZnAl₂O₄ spinels sintered up to 1000 °C.

Figure 5 illustrates the SEM micrographs and EDX analyses of the synthesized MnAl₂O₄ and ZnAl₂O₄ spinels. The SEM images of MnAl₂O₄ (Fig. 5a) show clusters of agglomerated particles with irregular shapes and a relatively compact surface. The interconnected grains reflect the typical microstructural features of spinel ceramics produced via solid-state sintering. The corresponding EDX spectrum confirms the expected elemental composition, displaying clear peaks of Mn, Al, and O without detectable impurities. Quantitative EDX values further indicate that Mn is the dominant element, in agreement with the MnAl₂O₄ stoichiometry, confirming the successful formation of the manganous aluminate spinel^30^. In contrast, the SEM images of ZnAl₂O₄ (Fig. 5b) exhibit a more granular and highly textured surface, composed of fine and densely packed particles characteristic of the gahnite spinel formed through solid-state synthesis. The EDX spectrum reveals distinct peaks for Zn, Al, and O, verifying the chemical purity of the ZnAl₂O₄ phase. Quantitative analysis shows Zn, Al, and O in their expected ratios, supporting the formation of a single-phase gahnite structure^31^. Collectively, the SEM–EDX results confirm the successful synthesis of pure, well-crystallized cubic spinel phases, consistent with the XRD.

### In vitro investigation

#### X-ray diffraction

**Fig. 6:**
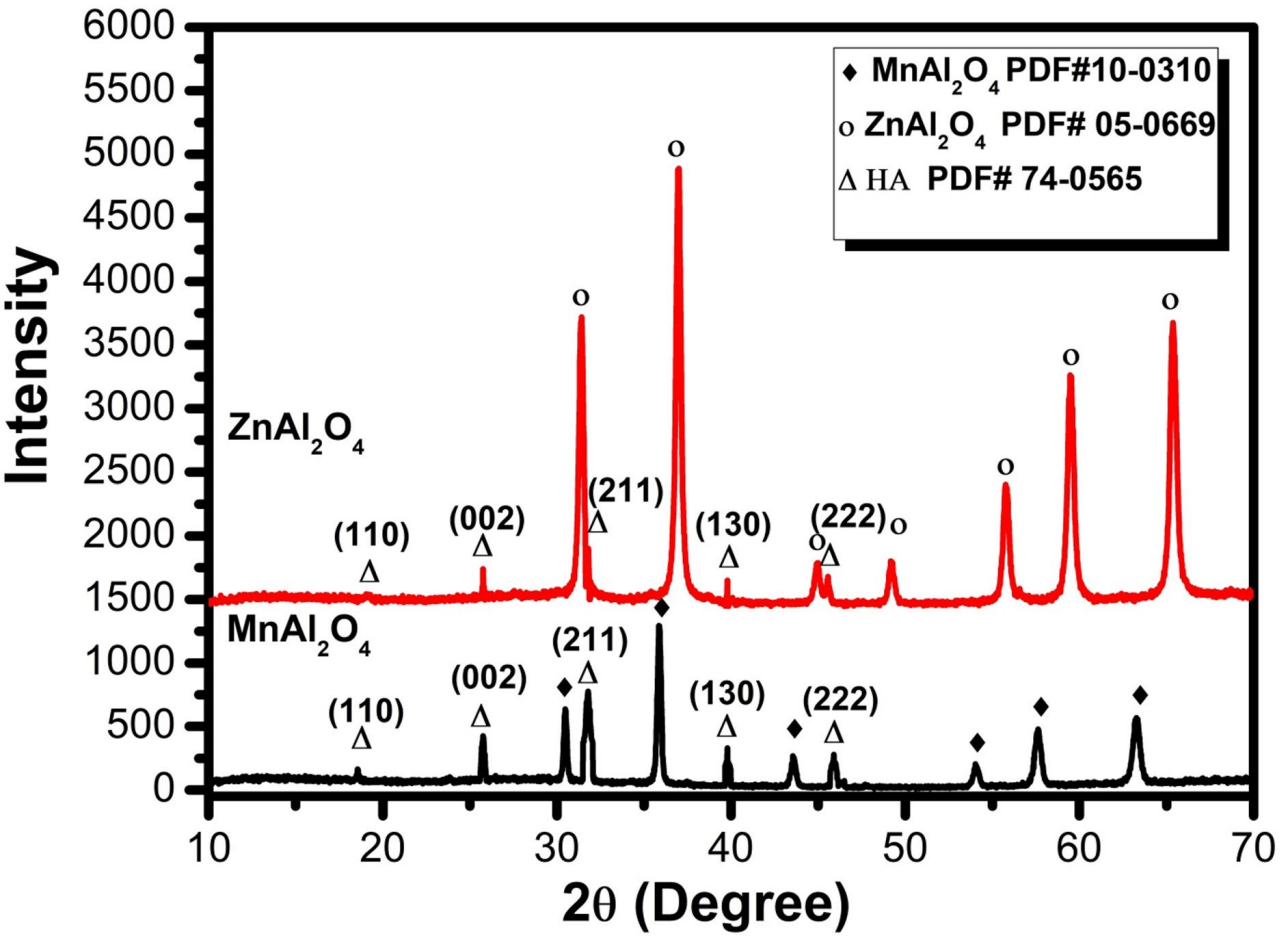
XRD patterns of MnAl₂O₄ and ZnAl₂O₄ spinel samples after 28 d of immersion in SBF.

Figure 6 presents the XRD profiles of MnAl₂O₄ and ZnAl₂O₄ spinels following 28 days of immersion in SBF, offering compelling evidence of surface-mediated bioactivity. Diffraction peaks corresponding to the characteristic hydroxyapatite planes (110), (002), (130), and (222) in accordance with PDF#74–0565 confirm the nucleation of an apatite layer on both material surfaces^36,37^. Notably, the HA signals are more pronounced in the MnAl₂O₄ sample, suggesting either enhanced crystallinity or a greater extent of apatite formation relative to its Zn-containing counterpart, where intense spinel reflections partially obscure the HA pattern^6,10^. While these findings affirm the bioactive potential of both spinels, the emerging diffraction pattern remains incomplete, indicative of partial surface coverage at this 28-day time point. This partial crystallinity is not a limitation but rather a functional advantage: partially crystalline HA is known to confer enhanced bioactivity and osteocompatibility, while the underlying spinel framework preserves mechanical integrity, yielding a composite architecture that synergistically balances biological performance with structural durability. To build upon these promising observations, future work will extend the immersion period to achieve full mineralization, yielding well-resolved, high-intensity HA reflections that unequivocally confirm complete apatite maturation.

#### FTIR spectra

**Fig. 7:**
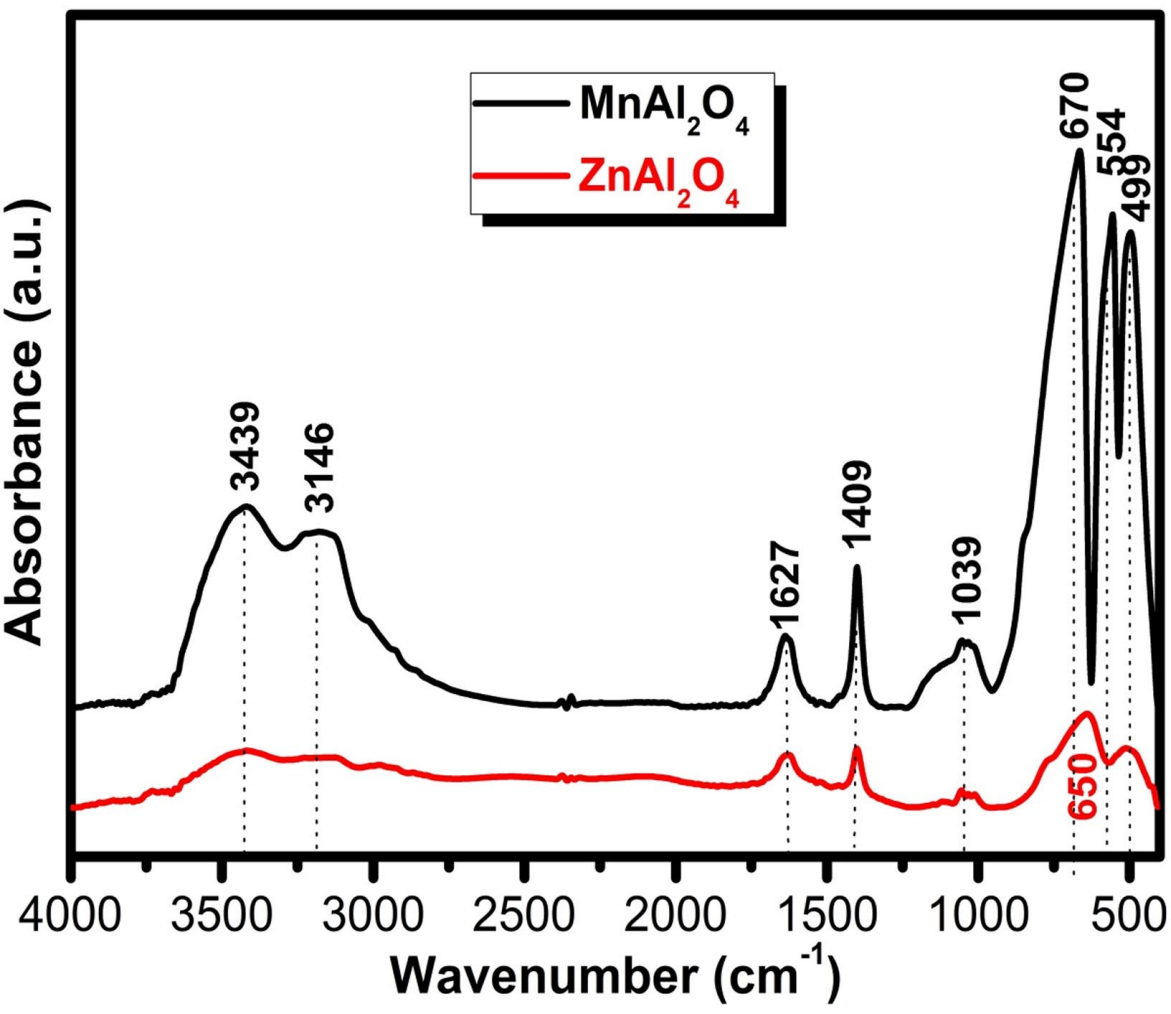
FTIR spectra of MnAl₂O₄ and ZnAl₂O₄ spinel samples after 28 d of immersion in SBF.

Figure 7 shows the infrared spectra of MnAl₂O₄ and ZnAl₂O₄ spinel samples after immersion in simulated body fluid (SBF), highlighting the chemical changes that occur on their surfaces during the soaking period. Both samples exhibit new or intensified absorption bands typically associated with apatite formation, indicating bioactivity. For both samples, several strong peaks appear in the 1400–1500 cm⁻¹ region, which correspond to carbonate (CO₃²⁻) vibrations, suggesting the formation of carbonated hydroxyapatite on its surface^37^. Additionally, bands around 1100–1000 cm⁻¹ and 650–560 cm⁻¹, reflecting phosphate group incorporation due to apatite nucleation^6,10,38^.

The FTIR analysis after SBF immersion provides direct evidence of bioactivity for both MnAl₂O₄ and ZnAl₂O₄. The appearance of characteristic phosphate and carbonate bands confirms the formation of a bone-like carbonated hydroxyapatite layer on each spinel’s surface. Significantly, the more pronounced spectral evolution for MnAl₂O₄ indicates a faster and more robust apatite formation compared to ZnAl₂O₄, highlighting a clear difference in their bioactive kinetics and surface reactivity.

**Fig. 8:**
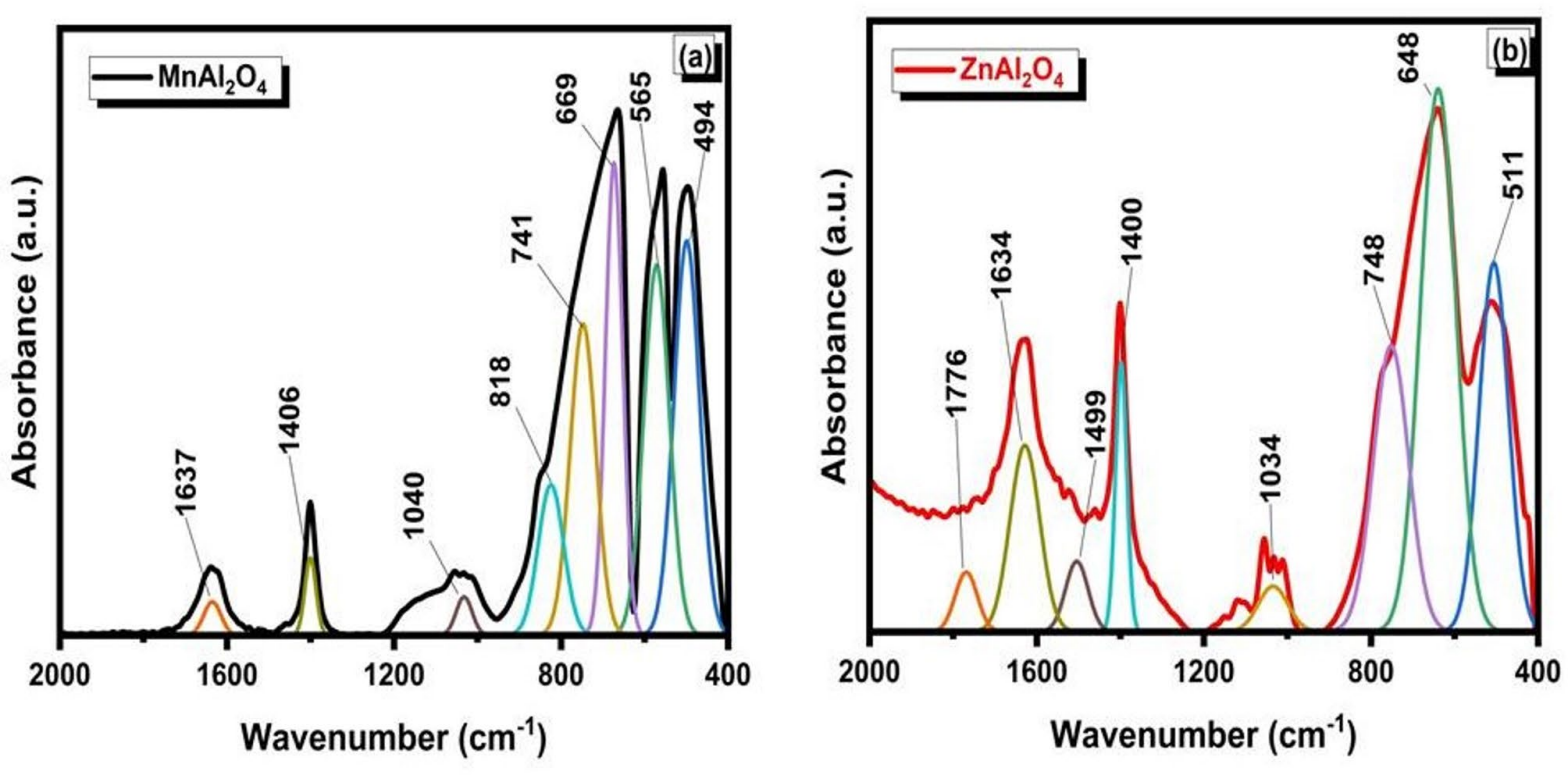
Deconvoluted FTIR spectra of (**a**) MnAl₂O₄ and (**b**) ZnAl₂O₄ spinel samples after immersion in SBF.

Figure 8 shows the deconvoluted FTIR spectra of (a) MnAl₂O₄ and (b) ZnAl₂O₄ after immersion in simulated body fluid (SBF), revealing the surface chemical changes associated with in-vitro bioactivity. For MnAl₂O₄, new and intensified bands appear in the higher wavenumber region at ~ 1040 cm⁻¹ and at 565 cm^-1^ corresponds to P–O stretching vibrations of phosphate groups (PO₄³⁻), confirming apatite layer formation^6,39^. Additionally, the band around ~ 1637 and ~ 1406 cm⁻¹, which are attributed to the bending vibrations of adsorbed water and carbonate (CO₃²⁻) groups. The presence of carbonate bands indicates the formation of carbonated apatite, a key marker of bioactivity^37^. In the low-wavenumber region (≈ 400–800 cm⁻¹), the metal–oxygen bands remain but show slight shifts and intensity changes (e.g., ~ 818, 741, 669, 565, and 494 cm⁻¹), suggesting surface interaction between the spinel lattice and the SBF ions^6^. In contrast, ZnAl₂O₄ exhibits weaker phosphate-related bands after immersion. Although band at ~ 1034 cm⁻¹ (P–O stretching), its intensity is noticeably lower than that observed for MnAl₂O₄. The low-frequency Zn–O and Al–O vibrations (~ 748, 648, and 511 cm⁻¹) show limited modification, indicating a comparatively reduced extent of surface apatite formation^10^.

The deconvoluted FTIR spectra after SBF immersion reveal a striking disparity in the bioactive response of the two spinels. MnAl₂O₄ exhibits strong, definitive phosphate and carbonate bands, confirming robust surface mineralization into a bone-like carbonated apatite layer, further supported by shifts in its intrinsic metal-oxygen lattice vibrations. In clear contrast, ZnAl₂O₄ displays only weak phosphate signatures with minimal lattice perturbation. This data conclusively demonstrates that MnAl₂O₄ possesses superior bioactivity, undergoing faster and more extensive surface-driven apatite nucleation than its zinc aluminate counterpart.

#### FE-SEM/EDX analysis

**Fig. 9:**
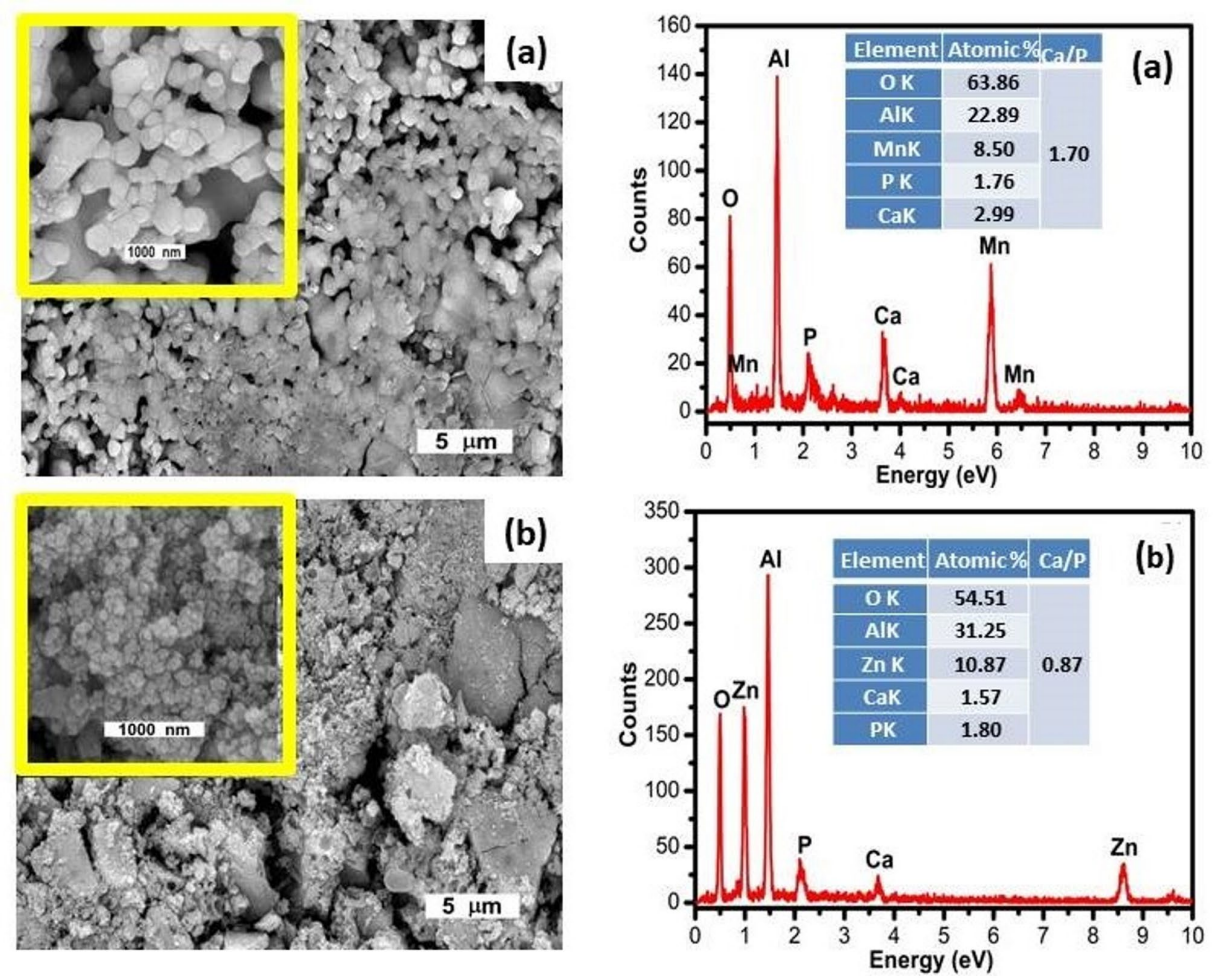
SEM micrographs and EDX of the synthesized (**a**) MnAl₂O₄ and (**b**) ZnAl₂O₄ spinel samples after immersion for 28d.

Figure 9 presents SEM micrographs and EDX spectra for MnAl₂O₄ and ZnAl₂O₄ spinel samples after immersion in SBF solution. The objective of these analyses is to investigate surface morphology and the extent of Ca–P deposition, which is a key indicator of bioactivity. The SEM images of MnAl₂O₄ (Fig. 9a) reveal a surface covered with fine, granular particles, indicating the nucleation and growth of Ca–P compounds on the spinel surface. The morphology suggests an active interaction between the MnAl₂O₄ surface and the ionic medium, promoting the formation of Ca–P clusters, which is typical for bioactive ceramic materials. The EDX spectrum further supports this observation. In addition to the characteristic peaks of Mn, Al, and O, the spectrum exhibits distinct signals corresponding to calcium (Ca) and phosphorus (P). Their presence indicates the formation of a Ca–P layer on the surface. The calculated Ca/P atomic ratio is approximately 1.70, which is close to that of hydroxyapatite (Ca/*P* = 1.67), suggesting that the deposited layer may be hydroxyapatite-like in nature^36,37^. This confirms the bioactive behavior of the MnAl₂O₄ spinel.

The SEM images of ZnAl₂O₄ **(**Fig. 9b) show a more porous and heavily textured surface with clusters of fine particles, indicating the deposition of Ca–P phases, though the coverage appears less dense than in MnAl₂O₄. This suggests a slightly lower nucleation rate compared to MnAl₂O₄ but still demonstrates clear surface reactivity. The EDX spectrum displays peaks corresponding to Zn, Al, and O, along with noticeable Ca and P signals. The Ca/P ratio is approximately 0.87, significantly lower than the stoichiometric hydroxyapatite ratio. This may indicate the early formation of amorphous calcium phosphate (ACP) or Ca-deficient Ca–P phase, which is typical in the initial stages of bio-mineralization^10^. This interpretation is further substantiated by the absence of distinct crystalline HA peaks in XRD and FTIR analyses after 28 days, indicating that only an initial Ca–P-rich layer had developed. As documented in the literature, such amorphous or calcium-deficient deposits are common precursors that may evolve into crystalline apatite under extended physiological conditions^40^. Additionally, the delayed or suppressed crystallization of HA on ZnAl₂O₄ may be attributed to the influence of Zn²⁺ ions released from the spinel surface, which are known to modulate apatite nucleation and growth kinetics, and in certain systems, can hinder the formation of a fully developed crystalline HA layer^41^.

SEM-EDX analysis delivers compelling evidence of both spinels’ bioactivity, revealing a striking contrast in their mineralization efficacy. While both MnAl₂O₄ and ZnAl₂O₄ successfully initiated calcium-phosphate (Ca–P) deposition, MnAl₂O₄ outperformed its counterpart, forming a near-ideal apatite layer with a Ca/P ratio of 1.70, which is closely matches that of stoichiometric hydroxyapatite (1.67). In marked contrast, ZnAl₂O₄ fostered the nucleation of earlier-stage, calcium-deficient precursors, evidenced by a Ca/P ratio of just 0.87. This divergence underscores a fundamental principle: even within the same crystal family, subtle variations in surface chemistry can tune the extent and maturity of bio-mineralization, offering a clear blueprint for designing next-generation bioactive ceramics with precise control over biological integration.

#### Bulk density and apparent porosity

**Fig. 10:**
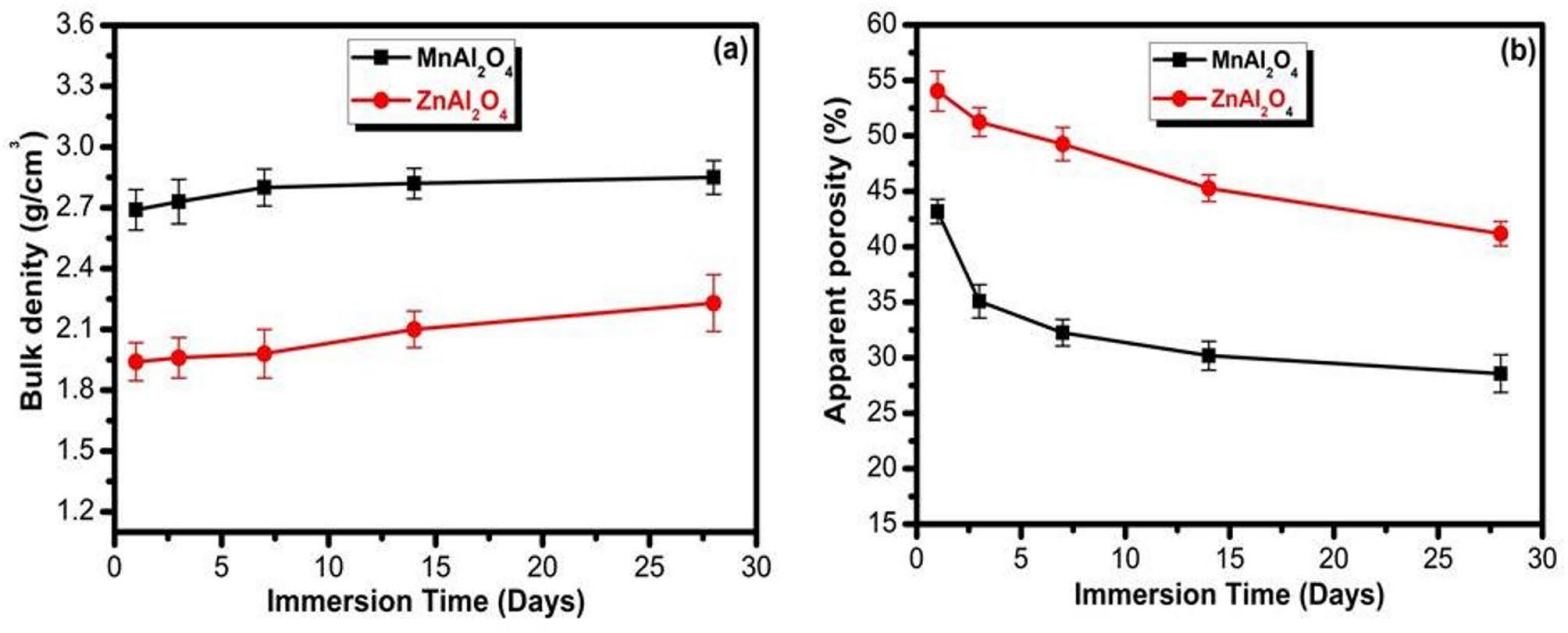
Bulk density and apparent porosity of MnAl_2_O_4_ and ZnAl_2_O_4_ spinal samples after immersion for 1, 3, 7, 14, and 28 d. The data are shown as means ± SD of three independent experiments.

Figures 10a-b illustrate how immersion time in simulated body fluid (SBF) affects the bulk density and apparent porosity of MnAl₂O₄ and ZnAl₂O₄ spinel samples. As immersion time increases, both materials show a gradual rise in bulk density (Fig. 10a), which indicates progressive filling of surface pores and internal voids by newly formed apatite or mineral deposits from SBF. This mineral deposition leads to a more compact microstructure, with MnAl₂O₄ consistently exhibiting higher density values than ZnAl₂O₄, reflecting its inherently denser structure and possibly faster mineral nucleation. Correspondingly, the apparent porosity (Fig. 10b) decreases steadily for both materials over time, confirming that SBF immersion results in pore occlusion due to hydroxyapatite precipitation^36,37^. MnAl₂O₄ shows a sharper porosity reduction, dropping from 43.17%*±* 1.1 to 28.58% *±* 1.7, which suggests more efficient pore filling and higher bioactivity compared with ZnAl₂O₄, whose porosity decreases more gradually from about 54.03%*±* 1.8 to 41.18%*±* 1.1. Overall, the trends demonstrate that longer SBF immersion enhances densification and reduces porosity of both spinels due to sustained surface mineralization, with MnAl₂O₄ showing a more pronounced response.

#### Compressive strength

**Fig. 11:**
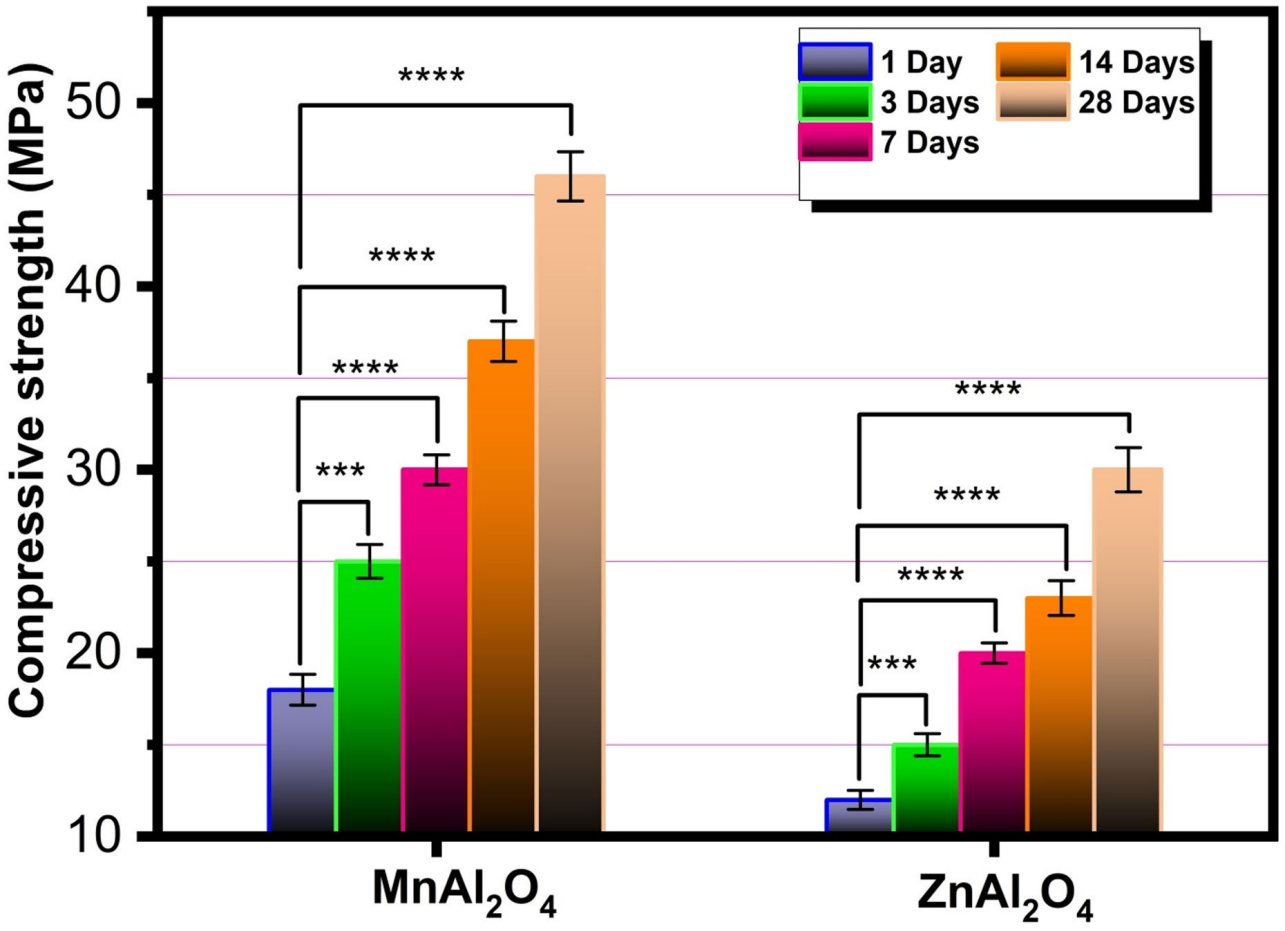
Compressive strength of MnAl_2_O_4_ and ZnAl_2_O_4_ spinal samples after immersion for 1, 3, 7, 14 and 28 d. The data are shown as means ± SD of three independent experiments. Significantly distinct at probability levels ***P ≤ 0.001 and ****p ≤ 0.0001.

Figure 11 shows the compressive strength of MnAl₂O₄ and ZnAl₂O₄ spinel samples after immersion in SBF for different periods (1, 3, 7, 14, and 28 d). For both materials, the compressive strength increases steadily with immersion time, reflecting progressive deposition of apatite layers and the gradual filling of surface pores by mineral phases from SBF. MnAl₂O₄ exhibits higher compressive strength at all-time points, rising from about 18 *±* 0.84 MPa on day 1 to 46 *±* 1.34 MPa after 28 days, indicating stronger structural integrity and faster mineralization. ZnAl₂O₄ shows the same trend but with lower absolute values, increasing from roughly 12 *±* 0.52 MPa to 30 *±* 1.21 MPa over the same period, suggesting a slower mineral growth rate and a less compact final structure. It has been demonstrated that increasing the compressive strength may be related to the decrease in porosity. The progressive increase in compressive strength observed for both spinels is far from incidental; it is the direct outcome of two synergistic and causally linked mechanisms. The first is intrinsic: a reduction in apparent porosity drives mechanical reinforcement through ceramic densification, as supported by prior work^30,31,42^. The second is interfacial: the concurrent deposition of a bone-like apatite layer during SBF immersion actively contributes to strength gain^36,37^. This biogenic apatite functions not merely as a surface deposit, but as a reinforcing phase sealing defects, bridging microcracks, and forming a cohesive composite interface with the underlying ceramic. The causal pathway is unambiguous: surface reactivity initiates apatite nucleation; the growing mineral layer densifies the surface zone; and this densification, in turn, elevates compressive strength. MnAl₂O₄ exemplifies this causality with striking clarity. Its nanoscale architecture and intrinsically reactive surface chemistry do not simply accelerate mineralization, they yield a thicker, more uniform, and tenaciously adherent apatite layer, culminating in markedly superior and more durable mechanical reinforcement. In essence, what we observe is not correlation, but causation etched in mineral and measured in MPa. While compressive strengths below 50 MPa may appear modest next to dense ceramic grafts, three perspectives reframe this discrepancy: First, our values stem from deliberately porous, bioactive scaffolds, not dense, non-porous implants that sacrifice bioactivity for strength. Second, the remarkable finding is not the absolute strength but the 42% gain over 28 days, demonstrating functional bioresilience ideally suited for moderate-load defects where progressive strengthening supports healing. Third, unlike dry testing that often overestimates performance, our values were measured after physiological immersion in SBF, a clinically relevant condition. Thus, our material prioritizes biological function over static mechanical density^30,42^.

#### Variation of pH and ions release

**Fig. 12:**
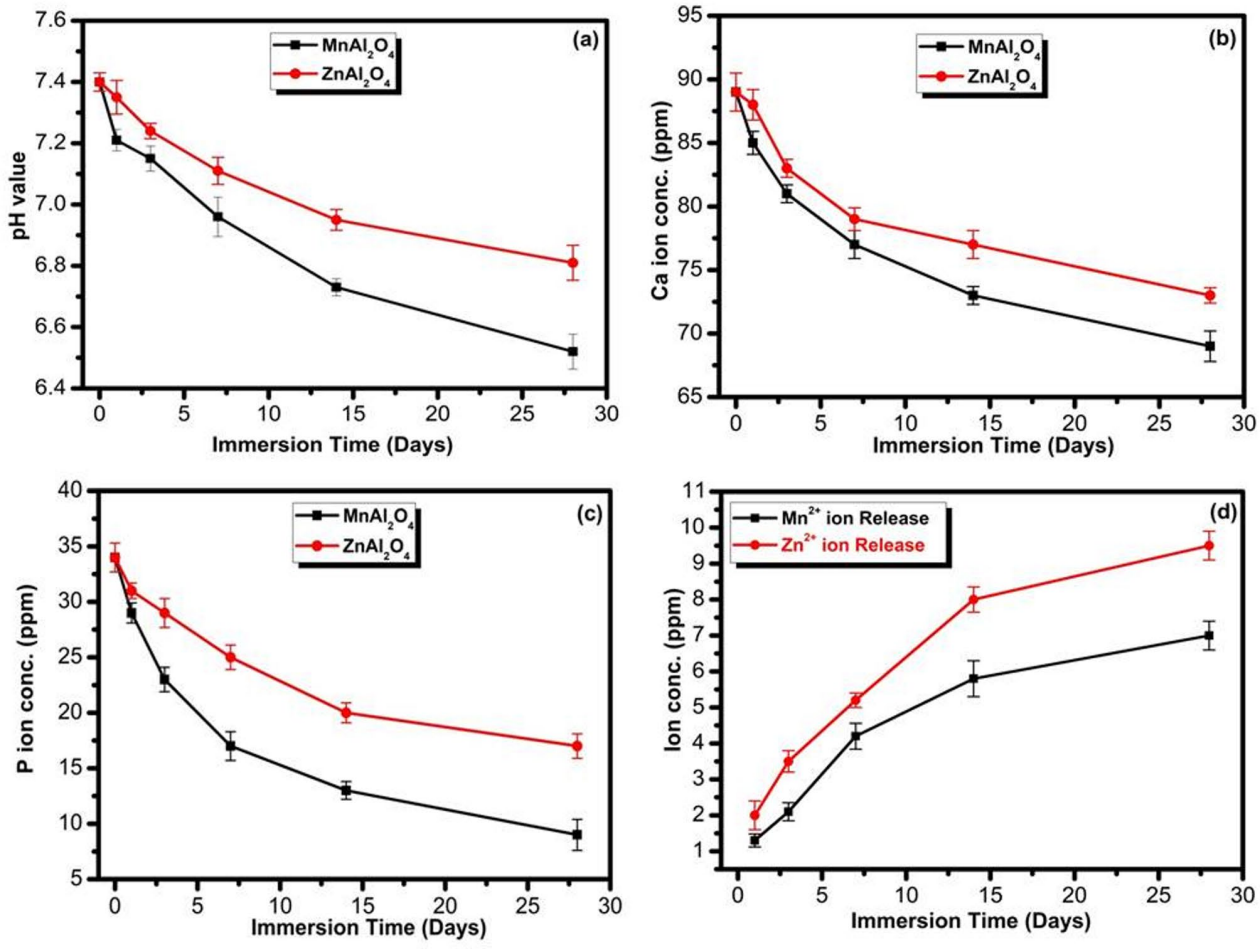
Variation of **(a)** pH, **(b)** Ca ion conc., **(c)** P ion conc., and (**d**) Mn & Zn ions conc. release of MnAl_2_O_4_ and ZnAl_2_O_4_ spinal samples after immersion for 1, 3, 7, 14, and 28 d. The data are shown as means ± SD of three independent experiments.

The dynamic evolution of the simulated body fluid (SBF) environment, detailed in Fig. 12, provides quantitative and compelling evidence for the superior bioactivity of MnAl₂O₄. Over the 28-day immersion period, both spinels induced the characteristic changes associated with surface reactivity and apatite nucleation: a gradual decrease in solution pH and a depletion of calcium (Ca²⁺) and phosphorus (P⁵⁺) ions. However, the kinetics and extent of these changes reveal a striking disparity between the two materials.

MnAl₂O₄ demonstrated a dramatically more aggressive interaction with the physiological medium. Its SBF exhibited a significantly sharper drop in pH **(**Fig. 12a**)**, signaling stronger ionic exchange and surface dissolution. Concurrently, MnAl₂O₄ caused a far more pronounced depletion of both Ca²⁺ and P⁵⁺ ions from the solution **(**Fig. 12b, c**)**, directly reflecting a rapid and substantial uptake of these building blocks for mineralization. This triad of accelerated changes; acidification coupled with intense ion consumption, conclusively points to faster nucleation and growth of a calcium-phosphate (apatite) layer on the MnAl₂O₄ surface^36,37^.

Figure 12d shows that both Mn²⁺ and Zn²⁺ ions are progressively released over 28 days, following a typical dissolution-controlled behavior with an initial increase followed by a gradual stabilization. Zn²⁺ exhibits a consistently higher cumulative release compared to Mn²⁺. In contrast, Mn²⁺ demonstrates a more controlled and moderate release profile, which may contribute to sustained biological performance and improved safety. However, it is important to note that Zn²⁺ has a narrow therapeutic window, meaning excessive release could induce cytotoxic effects^12^. In contrast, Mn²⁺ shows a more controlled release profile, which may provide safer and more sustained biological performance^43^.

In clear contrast, the changes induced by ZnAl₂O₄ were markedly slower and less intense across all three parameters, indicating a lower overall surface reactivity and a more subdued apatite-forming ability. Together, these kinetic profiles do not merely suggest a difference in degree but highlight a fundamental divergence in bioactive potential, unequivocally confirming MnAl₂O₄ as the more reactive and promising candidate for bone-bonding applications.

#### Cytotoxicity result

**Fig. 13:**
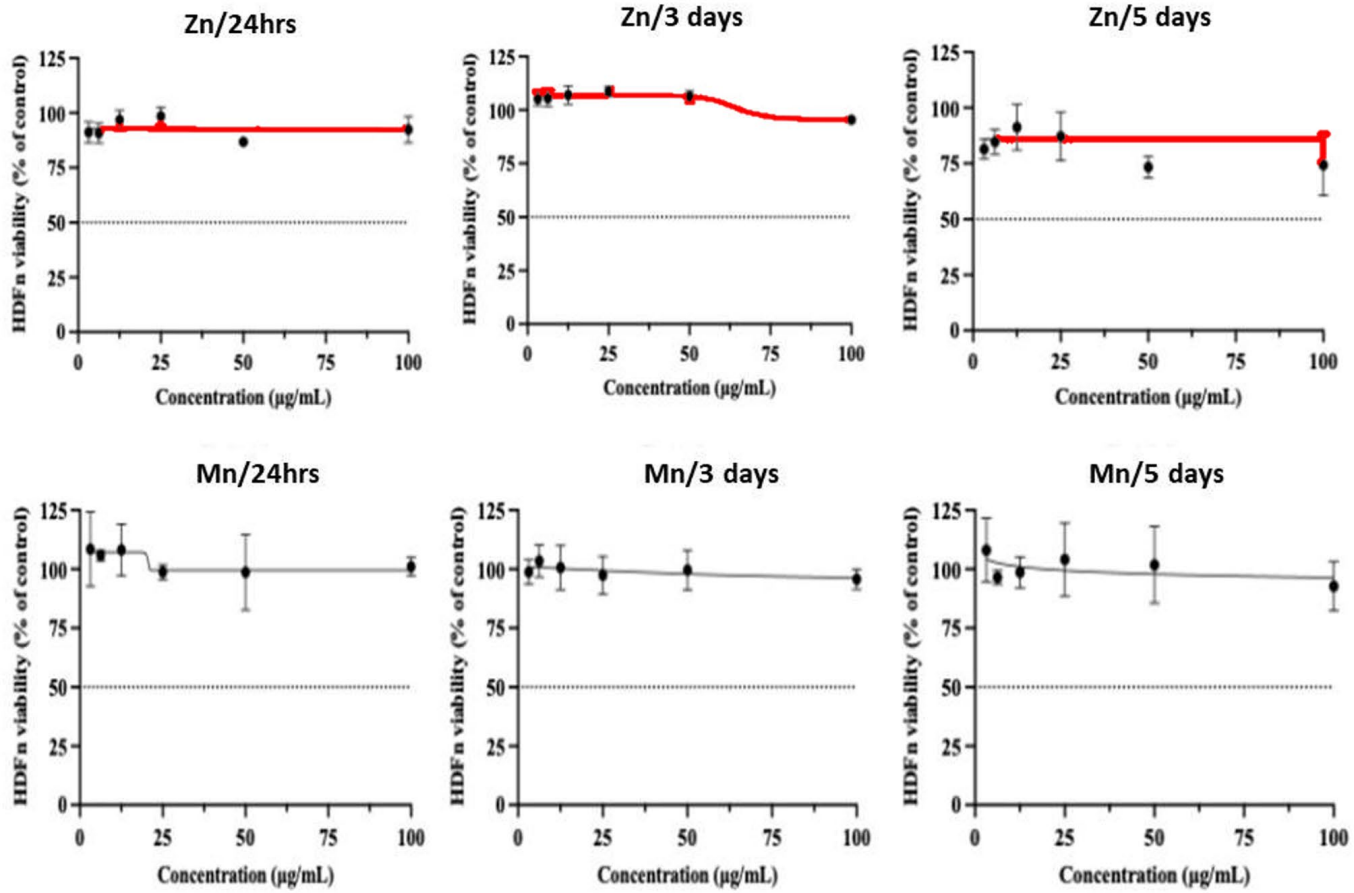
The dose-response curves of the samples on the HDFn cells after 1, 3, and 5 days. No more than 25% cell death was observed after 5 days at the highest tested concentration (100 µg/mL) in all samples. The data are shown as means ± SD of three independent experiments.

The MTT proliferation assay revealed pronounced differences in the long-term biocompatibility of the two spinel compositions (Fig. 13). MnAl₂O₄ exhibited excellent cytocompatibility at all tested concentrations and time points, with primary cells maintaining normal proliferative activity throughout the culture period. In contrast, ZnAl₂O₄, although initially well tolerated up to day 3, produced a reproducible 20% decline in cell viability by day 5 across all concentrations. These biological findings correlate closely with the respective ion degradation behaviors of Mn and Zn **(**Fig. 12d**)**. Importantly, this discrepancy is mechanistically meaningful rather than merely quantitative. We acknowledge that manganese, similar to zinc, functions within a narrow biological window beneficial at trace levels but potentially harmful under prolonged or excessive exposure^43,44^. However, the dissolution profile differs markedly between the two systems. Zn²⁺ shows a higher cumulative release over time, whereas Mn²⁺ displays a more controlled pattern characterized by an initial increase followed by gradual stabilization. The sustained and moderate release of Mn²⁺ likely underpins its improved cytocompatibility and safety profile. By contrast, the narrower therapeutic window of Zn²⁺ implies that excessive or sustained release may contribute to the observed cytotoxic effects^12^.

Moreover, a fundamental conceptual distinction must be emphasized: while manganese and zinc have been extensively investigated as dopants in resorbable systems such as hydroxyapatite, their incorporation into crystallographically inert aluminate frameworks represents an entirely different materials paradigm. In such systems, ion mobility and bioaccessibility are governed by spinel chemistry, not dopant diffusion, a mechanistic divergence that warrants dedicated investigation^40^. Thus, while MnAl₂O₄ emerges as a compelling candidate from this initial screening, its translational potential must ultimately be anchored in rigorous, mechanism-driven validation of ion behavior and long-term biological fate.

**Fig. 14:**
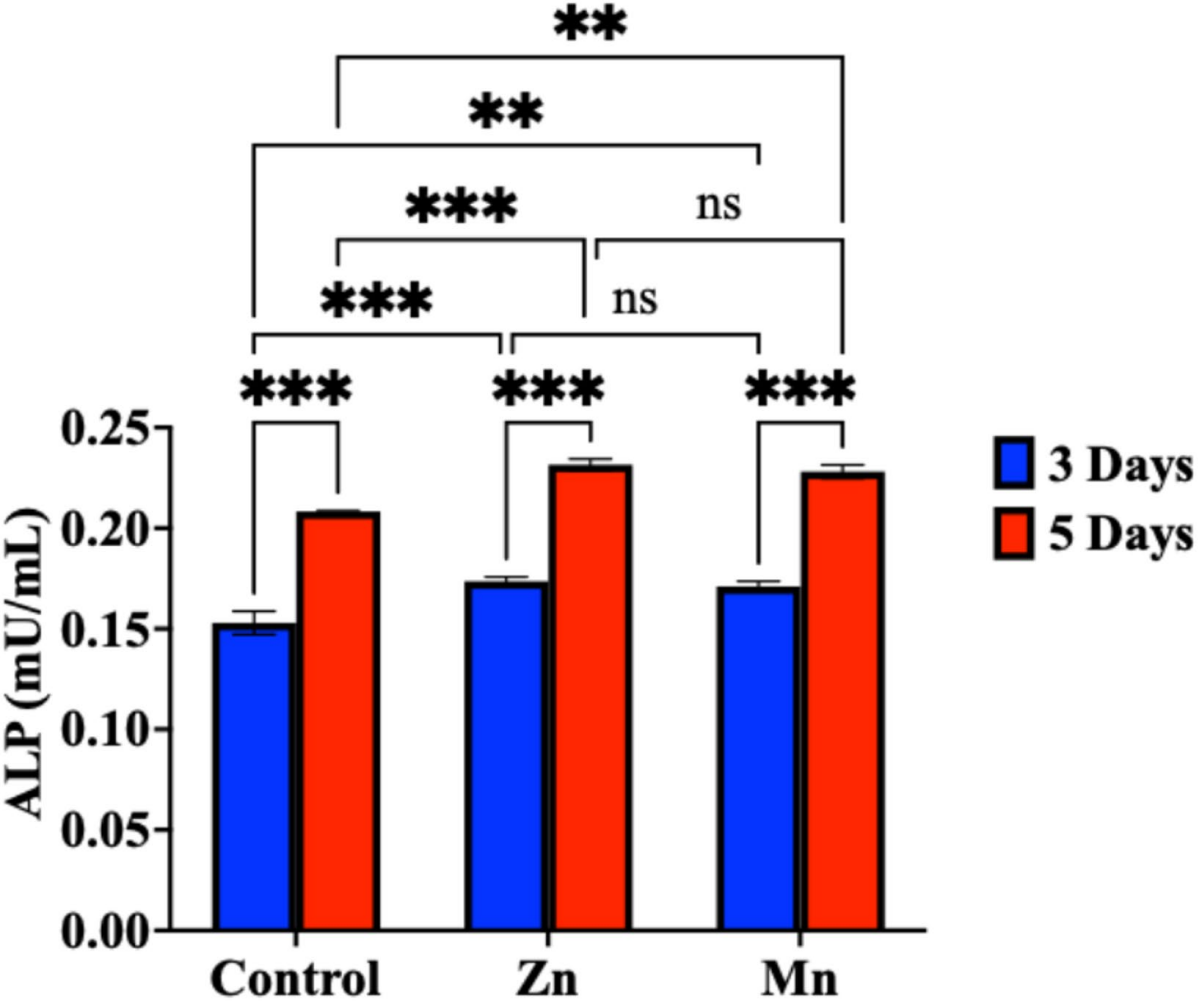
The ALP levels (mU/mL) secreted in the HDFn cells after 3 and 5 days. A 2-way ANOVA followed by Tukey’s multiple comparisons at a 95% confidence interval showed a significant increase in ALP after 5 days compared to after 3 days. The data are shown as means ± SD of three independent experiments. There is no significant difference in the ALP levels between Mn and Zn after 5 days. ** (*p* < 0.01), *** (*p* < 0.001), ns (*p* > 0.05).

ALP levels were measured at 3 and 5 days to assess the proliferative potential of the samples. It was apparent that ALP levels were enhanced over time. By comparing ALP levels between samples and over time, the Mn and Zn samples significantly enhanced the ALP levels compared to the control cells after 3 and 5 days. On comparing the Mn and Zn together, although the ALP was high, there was no significant difference between the two metals over time (Fig. 14).

**Fig. 15:**
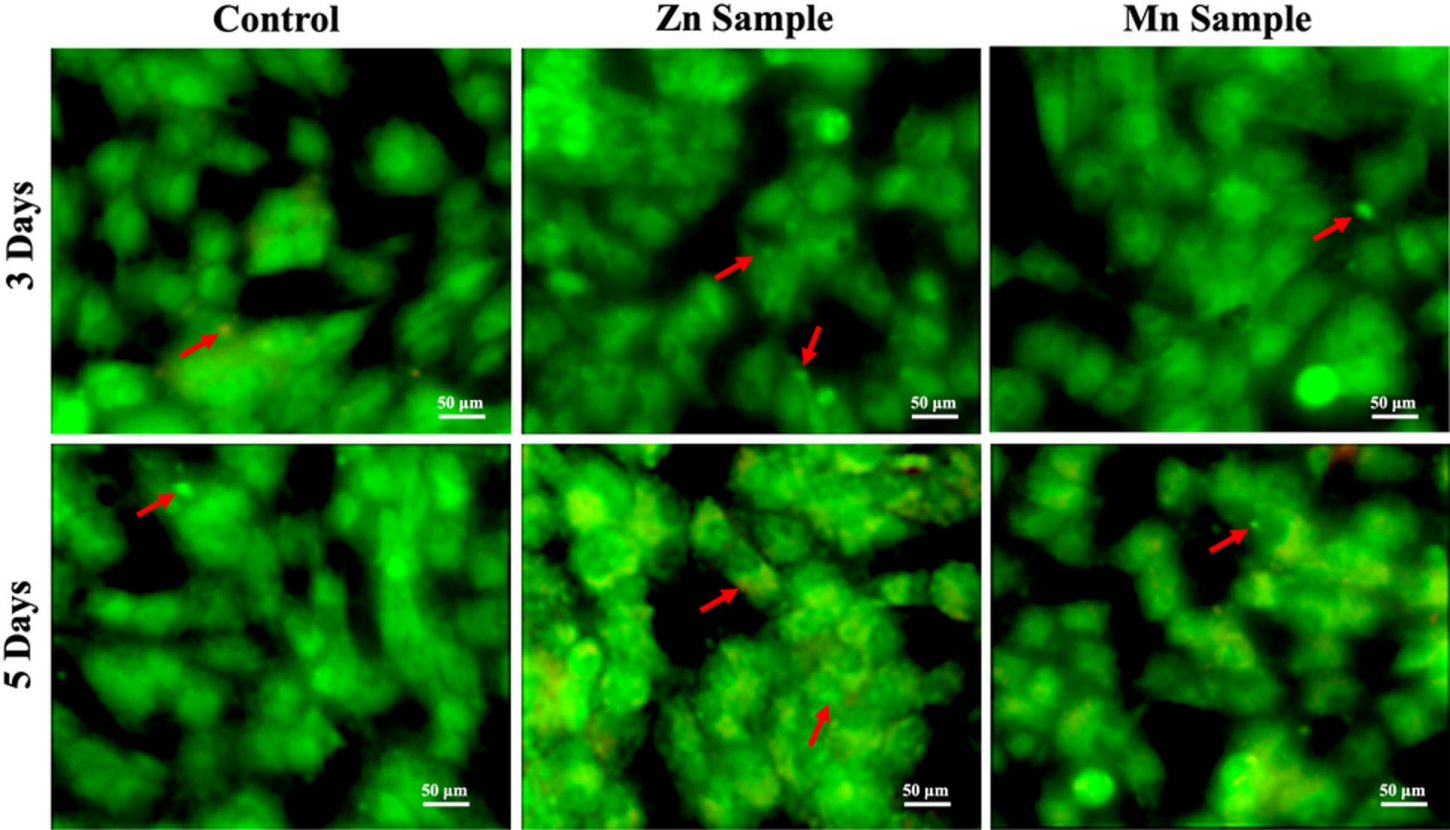
The fluorescent images of the HDFn cells treated with 50 µg/mL of samples after 3 and 5 days using the AO/EtBr stain. After 3 and 5 days, the cells remained intact, with compact nuclei and no shrinkage or deformities. Blebbing in the nuclei (red arrows) was also seen in the samples. After 5 days, the Mn sample showed apoptotic changes with orange cytoplasm. The magnification is 20X, and the scale bar is 50 μm.

The AO/EtBr stain under a fluorescent microscope revealed high cell compatibility with the samples *(*Fig. 15). After 3 and 5 days of application, the cells kept their intact morphologies, intact cell membranes, and normally condensed nuclei. Cell blebbing was uniform and within normal limits in all samples, including the control cells. The Mn sample after 5 days showed initiation of apoptotic changes with increased nuclear blebbing.

**Fig. 16:**
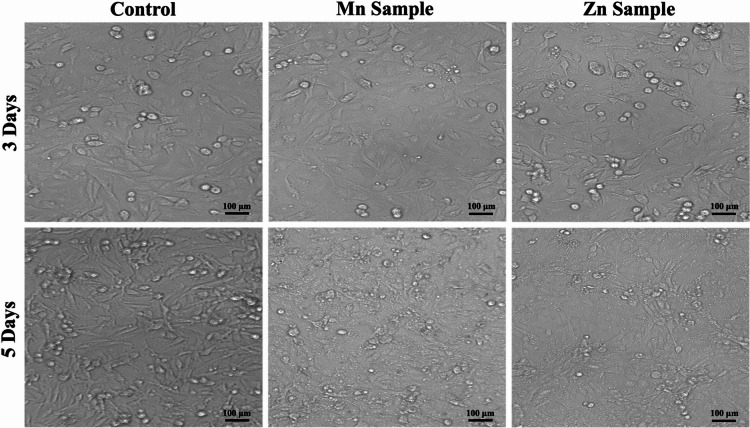
The light microscope photos of HDFn cells treated with 50 µg/mL of both samples after 3 and 5 days. The cells look uniform with normal growth and intact shape. Only the Mn sample after 5 days showed some deformity. The magnification is 10X, and the scale bar is 100 μm.

By tracing cell morphology under a light microscope **(**Fig. 16), there were no gross deformities in the cellular morphology. Cellular shape in the Zn sample was uniform over time, with enhanced growth. The Mn sample was uniform after one and three days, but after five days, some deformities in cell morphologies began to appear.

While both Mn- and Zn-based spinels demonstrated promising in-vitro biocompatibility by supporting cell viability, enhancing alkaline phosphatase (ALP) activity, and maintaining healthy morphology, their long-term biological profiles reveal a critical divergence. MnAl₂O₄ proved to be exceptionally biocompatible, showing no cytotoxicity across all tested doses and time points, and sustaining strong proliferative potential even during extended incubation, despite minimal apoptotic signs^45,46^. In contrast, ZnAl₂O₄ effectively stimulated early-stage osteogenic function, as evidenced by significantly enhanced ALP expression, but exhibited moderate, time-dependent cytotoxicity after five days^47,48^. This decisive contrast indicates that the manganese spinel is a robust candidate for long-term implantable applications, whereas the zinc aluminate holds considerable promise for short - to medium-term therapies where its potent bioactivity can be harnessed, provided its ionic release is precisely controlled to mitigate later-stage toxicity.

Our study positions MnAl₂O₄ as a promising preclinical candidate rather than a clinically ready material, having met first-line benchmarks necessary for further evaluation. The SBF bioactivity assay (Kokubo method) confirmed 7 days of apatite formation with near-stoichiometric Ca/P ratios, placing MnAl₂O₄ alongside established bioactive materials such as Bioglass^®^ and sintered hydroxyapatite. Cytotoxicity screening using fibroblasts, in accordance with ISO standards, demonstrated non-toxicity and serves as an initial safety assessment rather than a measure of osteogenic potential. Importantly, MnAl₂O₄ provides a combination of properties not typically observed in conventional bioactive ceramics: progressive mechanical reinforcement, evidenced by a ~ 42% increase in compressive strength over 28 days through apatite-mediated defect sealing, and controlled release of Mn²⁺ and Zn²⁺ ions that support osteogenic and angiogenic activity. While apatite nucleation occurs slightly slower than in hydroxyapatite or Bioglass^®^ (7 days vs. 1–3 days), the integration of sustained bioactivity, mechanical evolution, and therapeutic ion delivery positions MnAl₂O₄ as a complementary material for moderate-load bone graft applications, bridging bioactivity and structural support in preclinical settings^49,50^.

## Conclusions

This study successfully establishes a sustainable and scalable route to phase-pure MnAl₂O₄ and ZnAl₂O₄ ceramic spinels, with MnAl₂O₄ distinctly emerging as the standout candidate for bone regeneration. Its biomedical relevance is substantiated by a robust and converging body of evidence: (i) the rapid formation of a continuous, bone-like apatite layer within just 7 days of SBF immersion, affirming high surface bioactivity; (ii) a substantial 42% increase in compressive strength over 28 days, driven by apatite-induced surface densification and defect sealing; (iii) complete cytocompatibility with primary osteoblast-like cells across all tested concentrations and time points, confirming the absence of cytotoxic leachable; and (iv) retained structural integrity and phase purity post-immersion, underscoring long-term functional reliability. Collectively, these findings satisfy key preclinical benchmarks for bone graft materials, namely bioactivity, osteoconductive potential, mechanical compatibility, and biosafety. MnAl₂O₄ thus transcends descriptive bioactivity and enters the domain of validated functional bioresilience. Its nanoscale morphology and intrinsically reactive surface chemistry are not merely advantageous; they are mechanistically linked to each performance outcome. In contrast, ZnAl₂O₄, while osteoactive, exhibits time-dependent cytotoxicity beyond 3 days, limiting its utility to short-term or tightly regulated applications such as antibacterial coatings or temporary barriers. Ultimately, these results do more than advance the green synthesis of functional spinels; they deliver a stratified, evidence-based materials strategy for regenerative medicine: MnAl₂O₄ for load-sharing osseous repair requiring sustained integration, and ZnAl₂O₄ for controlled, transient interventions where bioactivity is harnessed within a defined therapeutic window.

## Data Availability

The datasets generated and/or analyzed during the current study are not publicly available because they are private, but are available from the corresponding author on reasonable request.
